# High homocysteine is associated with idiopathic normal pressure hydrocephalus in deep perforating arteriopathy: a cross-sectional study

**DOI:** 10.1186/s12877-023-03991-2

**Published:** 2023-06-21

**Authors:** Shisheng Ye, Kaiyan Feng, Yizhong Li, Sanxin Liu, Qiaoling Wu, Jinwen Feng, Xiaorong Liao, Chunmei Jiang, Bo Liang, Li Yuan, Hai Chen, Jinbo Huang, Zhi Yang, Zhengqi Lu, Hao Li

**Affiliations:** 1 Department of Neurology, Maoming People’s Hospital, Maoming, China; 2 Department of Radiology, Maoming People’s Hospital, Maoming, China; 3 Department of Neurology, the third affiliated hospital of Sun Yat-sen University, Guangzhou, China; 4 Department of Neurology, Maoming maternal and child health Hospital, Maoming, China

**Keywords:** Homocysteine, Idiopathic normal pressure hydrocephalus, Deep perforating arteriopathy, Cerebral small vessel disease, Mechanism

## Abstract

**Background and objective:**

The pathogenesis and pathophysiology of idiopathic normal pressure hydrocephalus (iNPH) remain unclear. Homocysteine may reduce the compliance of intracranial arteries and damage the endothelial function of the blood-brain barrier (BBB), which may be the underlying mechanism of iNPH. The overlap cases between deep perforating arteriopathy (DPA) and iNPH were not rare for the shared risk factors. We aimed to investigate the relationship between serum homocysteine and iNPH in DPA.

**Methods:**

A total of 41 DPA patients with iNPH and 49 DPA patients without iNPH were included. Demographic characteristics, vascular risk factors, laboratory results, and neuroimaging data were collected. Multivariable logistic regression analysis was performed to investigate the relationship between serum homocysteine and iNPH in DPA patients.

**Results:**

Patients with iNPH had significantly higher homocysteine levels than those without iNPH (median, 16.34 mmol/L versus 14.28 mmol/L; *P* = 0.002). There was no significant difference in CSVD burden scores between patients with iNPH and patients without iNPH. Univariate logistic regression analysis demonstrated that patients with homocysteine levels in the Tertile3 were more likely to have iNPH than those in the Tertile1 (OR, 4.929; 95% CI, 1.612–15.071; *P* = 0.005). The association remained significant after multivariable adjustment for potential confounders, including age, male, hypertension, diabetes mellitus, atherosclerotic cardiovascular disease (ASCVD) or hypercholesterolemia, and eGFR level.

**Conclusion:**

Our study indicated that high serum homocysteine levels were independently associated with iNPH in DPA. However, further research is needed to determine the predictive value of homocysteine and to confirm the underlying mechanism between homocysteine and iNPH.

## Introduction

Idiopathic normal pressure hydrocephalus (iNPH) is a treatable neurological disorder first described by Salomón Hakim in 1965 [1], characterized by the clinical triad of gait disturbance, cognitive deterioration, and urinary dysfunction in the absence of causative disorders, and radiological ventricular dilatation with normal CSF pressure on lumbar puncture [2]. iNPH is not a rare clinical entity. The estimated prevalence of iNPH is 1.4–3.7% in people aged 65 years and older, and increases with age [3]. Despite its relatively typical brain imaging and clinical symptoms, the pathogenesis and pathophysiology of iNPH are largely unknown. Currently, abnormal cerebrospinal fluid dynamics is often recognised as the underlying mechanism by reducing intracranial compliance [4–6].

Homocysteine is a sulfur-containing amino acid produced during methionine metabolism [7], which is known to be implicated in the pathogenesis of many clinical conditions, such as cerebral small vessel disease, stroke, and dementia [8–10]. It has been suggested that elevated homocysteine levels may reduce the compliance of intracranial arteries and damage the endothelial function of the BBB [5, 11–15], which may be the underlying mechanism of iNPH. One study showed that CSF homocysteine levels were significantly higher in iNPH patients compared with normal controls [16]. Ruxuan He et al. found that all patients with hydrocephalus were cobalamin C deficient, and all patients with cobalamin C deficiency had high homocysteine levels [17]. According to the above-mentioned evidence, we speculate that elevated homocysteine levels may be a risk factor for iNPH.

Deep perforating arteriopathy (DPA) is one of the most common forms of age-related cerebral small vessel disease (CSVD), causing cognitive impairment, lacunar infract and intracerebral haemorrhage [18]. In real-world practice, the overlapping prevalence of cases between DPA and iNPH are not rare, because of the shared risk factors (aging and vascular risk factors) [6, 19–21]. To the best of our knowledge, no study has been reported on the relationship between homocysteine and iNPH in DPA. In the present study, we aimed to investigate the relationship between serum homocysteine and iNPH in DPA.

## Materials and methods

### Study subjects

**Fig. 1:**
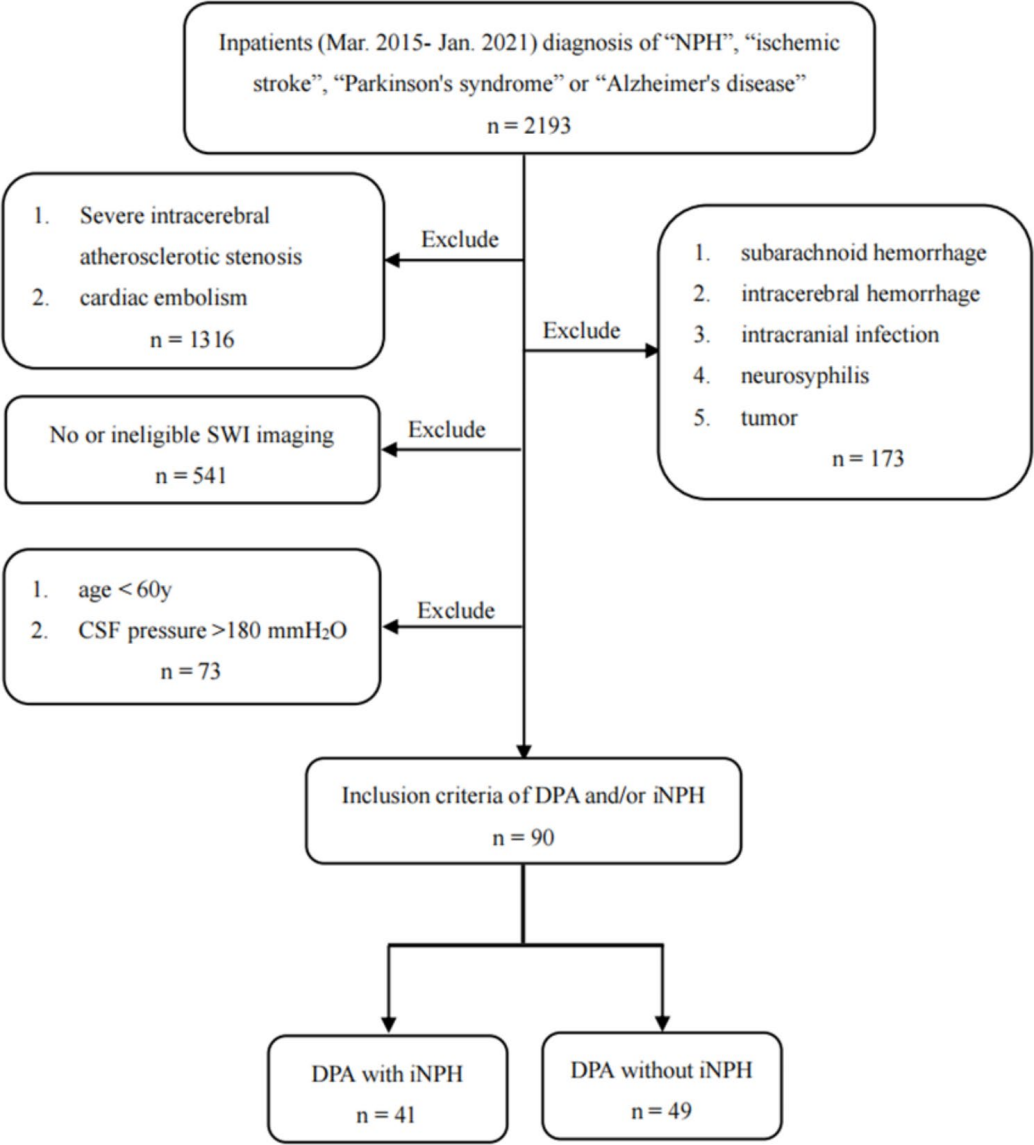
Flowchart of patient inclusion

This was a cross-sectional study. Between March 2015 and January 2021, a total of 2193 patients with NPH, ischemic stroke, Parkinson’s syndrome and Alzheimer’s disease were identified from the Department of Neurology of Maoming People’s Hospital and the Third Affiliated Hospital of Sun Yat-sen University. Among them, 90 patients finally fulfilled the inclusion criteria of DPA and/or iNPH (see “inclusion criteria of DPA and iNPH” for details). They were divided into two groups: DPA with iNPH group (n = 41), DPA without iNPH group (n = 49). The corresponding flowchart is shown in Fig. 1.

In addition, homocysteine levels were further subdivided into tertiles according to the number of patients and the distribution of homocysteine levels in order to observe whether improved performance could be quantified while maintaining a statistical effect in each category [9].

The study was approved by the Ethics Committee of Maoming People’s Hospital and Third Affiliated Hospital of Sun Yat-sen University.

### Inclusion criteria of DPA

(1) age ≥ 60 years; (2) at least one of the following atherosclerotic risk factors: smoking, alcohol drinking, BMI > 25, hypertension, diabetes mellitus, coronary heart disease, hyperlipidemia; (3) MRI neuroimaging met the STandards for ReportIng Vascular changes on nEuroimaging (STRIVE)-recommended standards [22]. All included patients were presented with deep microbleeds, including microbleeds in brain stem, dentate, basal ganglion, regardless of lobar cerebellar or lobar microbleeds. Exclusion criteria: (1) craniocerebral trauma; (2) intracerebral space-occupying lesions; (3) lesions in the central nervous system secondary to infectious, metabolic, immunological, toxic and tumorous causes; (4) ischemic stroke resulting from lager cerebral arteries occlusion, cardiac embolism; (5) severe intracerebral atherosclerotic stenosis that could alter cerebral hemodynamics; (6) intracerebral hemorrhage.

### Inclusion of iNPH

(1) age ≥ 60 years; (2) more than one of the clinical triad: gait disturbance, cognitive impairment, and urinary incontinence; (3) ventricular dilation (Evans’ index > 0.3); (4) CSF pressure of 180 mmH_2_O or less; (5) one of the following three investigational features: (a) neuroimaging features of narrowing of the sulci and subarachnoid spaces over the high convexity/midline surface under the presence of gait disturbance; (b) improvement of symptoms after CSF tap test; (c) improvement of symptoms after CSF drainage test; (6) exclude diseases that may cause ventricular dilation, including subarachnoid hemorrhage, meningitis, head injury, congenital hydrocephalus, and aqueductal stenosis; (7) above-mentioned clinical symptoms cannot be fully explained by other neurological or non-neurological diseases.

### MRI protocol and parameters

Patients underwent brain MRI on a GE 3.0-T scanner (Discovery MR750, General Electric, Milwaukee, USA) operated by research-dedicated technical staff. Sequences included axial T1 FLAIR weighted, axial T2 POPELLER weighted (FrFSE), T2 fluid-attenuated inversion recovery weighted (T2 FLAIR), axial 3-dimensional time of flight MR angiography (3D-TOF MRA) and axial T2*-weighted angiography (SWAN). Details of MRI protocol and parameters can be found in our previous article [23].

### CSVD burden assessment

According to the STRIVE recommendation, neuroimaging markers of CSVD include recent small subcortical infarcts, lacunes, white matter hyperintensities (WMH), perivascular spaces (PVS), cerebral microbleeds (CMBs), and brain atrophy [22]. Based on the ordinal “SVD score”, we rated the CSVD burden with a total score of 4, on the basis of the 4 MRI markers (lacunes, white matter hyperintensities, perivascular spaces, microbleeds) of CSVD [24]. One point was awarded for each of the following: presence of one or more lacunes, presence of one or more cerebral microbleeds, presence of moderate to severe (grade 2 ~ 4) PVS in basal ganglia, presence of periventricular WMH Fazekas 3 and/or deep WMH Fazekas 2 ~ 3. All images were independently rated by 2 vascular neurologists.

### Data collection

Demographic characteristics, vascular risk factors, laboratory results, and neuroimaging data were collected and recorded by trained research staff. Demographic characteristics included age, gender and body mass index (BMI). Vascular risk factors included smoking status (defined as continuous or cumulative smoking ≥ 6 months, or less than 6 months daily of smoking, including former smoking and current smoking), alcohol consumption (defined as average alcohol consumption ≥ 40 g/d, continuous or cumulative drinking ≥ 6 months), hypertension, diabetes mellitus, hypercholesterolemia, coronary heart disease, atrial fibrillation, previous ischemic stroke, and atherosclerotic cardiovascular disease (ASCVD). A history of hypertension, diabetes mellitus, hypercholesterolemia, coronary artery disease, and atrial fibrillation was based on documentation at admission and did not include a new diagnosis made during incident hospitalization. ASCVD is defined as any of the following: myocardial infarction or angina pectoris, coronary artery disease, ischemic stroke or transient ischemic attack, and peripheral arterial disease [25]. Laboratory tests including counts of neutrophil, lymphocyte and platelet were measured at time of admission. Total cholesterol (TC), triglycerides (TG), high-density lipoprotein cholesterol (HDL-C), low-density lipoprotein cholesterol (LDL-C), uric acid, creatinine, fasting plasma glucose (FPG), homocysteine and apolipoproteins E (APOE) genotype were measured in the next morning after admission. All the laboratory results were measured using standard laboratory methods. Neutrophil-lymphocyte ratio (NLR), platelet-lymphocyte ratio (PLR) and estimated glomerular filtration (eGFR) were calculated from above laboratory results. eGFR was calculated using the Chronic Kidney Disease Epidemiology Collaboration (CKD-EPI) equation for the Asian population [26].

### Statistical analysis

Data for continuous variables were reported as mean ± standard deviation or medians and interquartile range, depending on the normal or non-normal distribution of data tested by Shapiro-Wilk test, and categorical variables were reported as numbers with percentages. Comparisons were performed using the Pearson chi-square test or Fisher exact test, independent-samples t test, and Mann-Whitney U test for univariate analysis.

Comparison of multiple mean values between subgroups was conducted by one-way analysis of variance or Kruskal-Wallis H test as appropriate. Variables that were considered clinically relevant or with *P <* 0.15 in the univariate analysis were included in the multivariable logistic regression model-building process to determine factors for iNPH in DPA patients. Statistical analyses were performed using SPSS 25.0 (SPSS, Chicago, IL, USA). A *P* value < 0.05 was considered statistically significant (2-sided).

## Results

The average age of the enrolled patients was 70.4 ± 8.6 years. There were 68 (75.6%) males. The median of serum homocysteine was 15.5 mmol/L, and the tertiles of serum homocysteine were as follows: Tertile1, < 14.0 mmol/L; Tertile2, 14.0 to 16.9 mmol/L; Tertile3, ≥16.9 mmol/L. Table 1 shows that higher homocysteine levels were associated with male sex (*P* < 0.001), frequency of lobar CMBs (*P* = 0.006) and total CMBs (*P* = 0.004).

**Table 1:** Characteristics of patients according to serum homocysteine tertiles

Variables	Homocysteine (mmol/L)				
	Total	<14.0	14.0 -16.9	≥ 16.9	*P* Value
Age (y)	70.42 ± 8.63	69.00 ± 10.11	71.20 ± 8.01	71.07 ± 7.68	*P* = 0.547
Male, n (%)	68(75.6%)	14(46.7%)	26(86.7%)	28(93.3%)	*P*<0.001
Body mass index	22.7(20.8,24.2)	22.0 (20.8,24.0)	23.0 (20.8,23.9)	20.8 (21.7, 24.7)	*P* = 0.593
Alcohol drinking, n (%)	6(6.7%)	3 (10.0%)	1 (3.3%)	2 (6.7%)	*P* = 0.585
Smoking status					
Former, n (%)	5(5.6%)	3(10.0%)	1(3.3%)	1(3.3%)	*P* = 0.429
Current, n (%)	9(10.0%)	2(6.7%)	1(3.3%)	6(20.0%)	*P* = 0.075
Hypertension, n (%)	71(78.9%)	25(83.3%)	23(76.7%)	23(76.7%)	*P* = 0.766
Diabetes Mellitus, n (%)	22(24.4%)	6(20.0%)	6(20.0%)	10(33.3%)	*P* = 0.382
Coronary artery disease, n (%)	5(5.6%)	4(13.3%)	1(3.3%)	0(0%)	*P* = 0.064
Atrial fibrillation, n (%)	1(1.1%)	1(3.3%)	0(0%)	0(0%)	*P* = 0.364
Previous stroke, n (%)	33 (36.7%)	10(33.3%)	9(30.0%)	14(46.7%)	*P* = 0.366
ASCVD/hypercholesterolemia, n (%)	28 (31.1%)	8(26.7%)	9(30.0%)	11(36.7%)	*P* = 0.696
NLR	3.20 (2.15,4.51)	3.08 (2.09,5.23)	3.28 (2.42,4.22)	2.90 (1.81,3.70)	*P* = 0.665
PLR	155.50(98.73,215.65)	157.76(96.38,213.18)	160.78(112.87,235.98)	136.13(101.44,214.95)	*P* = 0.919
Triglycerides (mmol/L)	1.22 (0.88,1.73)	1.10 (0.87,1.60)	1.42 (0.87,1.94)	1.13 (0.94,1.66)	*P* = 0.416
Cholesterol (mmol/L)	4.59 ± 1.10	4.34 ± 0.97	4.83 ± 1.35	4.63 ± 0.92	*P* = 0.229
LDL-C (mmol/L)	2.73 (2.14,3.22)	2.56 (2.10,3.23)	2.79 (2.04,3.55)	2.83 (2.32,3.14)	*P* = 0.523
HDL-C (mmol/L)	1.15 (0.98,1.35)	1.10 (0.98,1.31)	1.15 (1.02,1.35)	1.15 (0.94,1.54)	*P* = 0.460
uric acid (mmol/L)	357.36 ± 108.68	324.26 ± 91.20	392.01 ± 119.43	348.97 ± 103.60	*P* = 0.071
eGFR ml/min/1.73m^2^	74.03 ± 18.73	79.58 ± 14.87	74.17 ± 17.74	68.53 ± 21.73	*P* = 0.075
FPG (mmol/L)	5.90 ± 2.11	5.85 ± 2.16	5.80 ± 1.87	6.06 ± 2.36	*P* = 0.893
APOE ε4, n (%)	11 (24.4%)	2 (22.2%)	5 (26.3%)	4(23.5%)	*P* = 0.967
Neuroimaging markers					
Lacunes, n	3 (1,7)	3 (1,7)	4 (1,7)	3.5 (1,6.25)	*P* = 0.817
Deep CMBs, n	4(1.5,13.5)	3(1,7)	3.5(1,16)	7.5 (3,16)	*P* = 0.075
Lobar CMBs, n	5 (2,11.5)	3 (1,5)	7 (0,17)	8 (3.75,18.25)	*P* = 0.006
Total CMBs, n	10.5 (5,26.5)	6 (4,11.5)	11.5 (2.75,38.5)	19.5 (7.75,32)	*P* = 0.004
PVS-BG, n	41.5 (28,51.0)	35 (15.5,50.5)	40 (30,52.75)	42 (30,59.75)	*P* = 0.241
WMH Fazekas ≥ 2	84 (93.3%)	28 (93.3%)	28 (93.3%)	28 (93.3%)	*P* = 1.00
Burden score	4 (3,4)	4 (3,4)	4 (3.75,4)	4 (4,4)	*P* = 0.502

ASCVD indicates atherosclerotic cardiovascular disease; NLR, neutrophil-lymphocyte ratio; PLR, platelet-lymphocyte ratio; eGFR, estimated glomerular filtration rate; HDL-C, high-density lipoprotein cholesterol; LDL-C, low-density lipoprotein cholesterol; FPG, fasting plasma glucose; APOE, apolipoproteins E; WMH, white matter hyperintensities; CMBs, cerebral microbleeds; PVS, perivascular spaces; BG, basal ganglia

Table 2 shows the demographic characteristics, vascular risk factors, laboratory results and radiographic images of DPA patients with and without iNPH. Patients with iNPH had a significantly higher homocysteine level (median, 16.34 mmol/L versus 14.28 mmol/L; *P* = 0.002) than those without iNPH. There was no significant difference in CSVD neuroimaging markers (lacunes, deep CMBs, lobar CMBs, total CMBs, PVS-BG, WMH Fazekas≥2) and CSVD burden scores between patients with iNPH and patients without iNPH. In univariate logistic regression analysis, those patients with iNPH were associated with older age (OR, 1.094; 95% CI, 1.033–1.159; *P* = 0.002), male gender (OR, 5.371; 95% CI, 1.644–17.547; *P* = 0.005), previous ischemic stroke (OR, 2.637; 95% CI, 1.092–6.369; *P* = 0.031), ASCVD/hypercholesterolemia (OR, 4.881; 95% CI, 1.843–12.929; *P* = 0.001), lower levels of eGFR (OR, 0.971; 95% CI, 0.946–0.996; *P* = 0.021), and higher levels of homocysteine (OR, 1.129; 95% CI, 1.028–1.240; *P* = 0.011).

**Table 2:** Characteristics of DPA patients with and without iNPH.

Variables	DPA + iNPH N = 41	DPA N = 49	Unadjusted OR (95% CI)	P Value
Age (y)	73.56 ± 8.95	68.75 ± 8.35	1.094 (1.033,1.159)	*P* = 0.002
Male, n (%)	37(90.2%)	31(63.3%)	5.371 (1.644,17.547)	*P* = 0.005
Body mass index	21.76 (20.81,23.75)	23.09 (20.59, 24.71)	0.904 (0.758,1.080)	*P* = 0.266
Alcohol drinking, n (%)	2(4.9%)	4(8.2%)	0.577 (0.100,3.322)	*P* = 0.538
Smoking status				
Former, n (%)	4(9.8%)	1(2.0%)	5.189 (0.556,48.397)	*P* = 0.148
Current, n (%)	5(12.2%)	4(8.2%)	1.563 (0.391,6.248)	*P* = 0.528
Hypertension, n (%)	32(79.6%)	39(78.0%)	0.912 (0.331,2.515)	*P* = 0.858
Diabetes Mellitus, n (%)	10(24.4%)	12(24.5%)	0.995 (0.379,2.612)	*P* = 0.991
Coronary artery disease, n (%)	1(2.4%)	4(8.2%)	0.281(0.030, 2.622)	*P* = 0.265
Atrial fibrillation, n (%)	0(0%)	1(2.0%)	0(0,0)	*P* = 1.000
Previous stroke, n (%)	20(48.8%)	13(26.5%)	2.637(1.092,6.369)	*P* = 0.031
ASCVD/hypercholesterolemia, n (%)	20(48.8%)	8(16.3%)	4.881 (1.843,12.929)	*P* = 0.001
NLR	3.30(2.25,4.37)	2.88(1.65,3.77)	1.012 (0.887,1.155)	*P* = 0.856
PLR	149.92(103.39,247.21)	152.63(93.19,200.18)	1.000 (0.995,1.004)	*P* = 0.904
Triglycerides (mmol/L)	1.28(0.73, 1.82)	0.99(0.79, 1.33)	1.620 (0.935,2.807)	*P* = 0.085
Cholesterol (mmol/L)	4.53 ± 1.05	4.65 ± 1.15	0.908 (0.618,1.334)	*P* = 0.622
LDL-C (mmol/L)	2.56(2.14, 2.95)	2.74(2.02, 3.39)	0.685 (0.404,1.161)	*P* = 0.160
HDL-C (mmol/L)	1.05(0.98, 1.24)	1.15(0.94, 1.35)	1.163 (0.379,3.565)	*P* = 0.792
Homocysteine (mmol/L)	16.34(14.82, 19.24)	14.28(11.73, 16.84)	1.129 (1.028,1.240)	*P* = 0.011
Uric acid (mmol/L)	371.73 ± 87.98	343.35 ± 125.17	1.003 (0.998,1.007)	*P* = 0.243
eGFR ml/min/1.73m^2^	68.93 ± 20.32	78.49 ± 16.15	0.971 (0.946,0.996)	*P* = 0.021
FPG, (mmol/L)	4.80(4.38, 6.27)	5.13(4.66, 6.77)	1.009 (0.821,1.240)	*P* = 0.932
APOE ε4, n (%)	7(25.0%)	4(23.5%)	1.083 (0.265,4.436)	*P* = 0.911
Neuroimaging markers				
Lacunes, n	3(1, 5.5)	3(1, 7)	0.944 (0.844,1.056)	*P* = 0.314
Deep CMBs, n	6(2, 16.5)	4(1.75, 10.75)	1.009 (0.981,1.039)	*P* = 0.523
Lobar CMBs, n	6(2.5, 16.5)	3(2, 10)	1.002 (0.982,1.021)	*P* = 0.865
Total CMBs, n	15(6.5, 33)	10(4, 19.25)	1.002 (0.990,1.015)	*P* = 0.712
PVS-BG, n	43(23, 59)	40.5(29.75, 48)	1.004 (0.986,1.002)	*P* = 0.675
WMH Fazekas ≥ 2	39 (91.8%)	45 (95.1%)	1.733 (0.301,9.982)	*P* = 0.538
Burden score	4(3, 4)	4(3.75, 4)	0.997 (0.507,1.961)	*P* = 0.994

ASCVD indicates atherosclerotic cardiovascular disease; NLR, neutrophil-lymphocyte ratio; PLR, platelet-lymphocyte ratio; eGFR, estimated glomerular filtration rate; HDL-C, high-density lipoprotein cholesterol; LDL-C, low-density lipoprotein cholesterol; FPG, fasting plasma glucose; APOE, apolipoproteins E; WMH, white matter hyperintensities; CMBs, cerebral microbleeds; PVS, perivascular spaces; BG, basal ganglia

Table 3 shows the results of multivariate analysis of the risk factors associated with iNPH. Univariate logistic regression analysis demonstrated that patients with homocysteine levels in Tertile3 were more likely to have iNPH compared with Tertile1 (OR, 4.929; 95% CI, 1.612–15.071; *P* = 0.005). The association remained significant after multivariable adjustment for potential confounders (tertile2: OR, 5.360; 95% CI, 1.341–21.427; *P* = 0.018; tertile3: OR, 6.055; 95% CI, 1.501–24.433; *P* = 0.011), including age, male, hypertension, diabetes mellitus, ASCVD or hypercholesterolemia, and eGFR level. The calibration of the model was good (Hosmer-Lemeshow goodness-of-fit *P* = 0.646).

**Table 3:** Multivariate analysis of the association between homocysteine and iNPH.

Homocysteine, mmol/L			Unadjusted model		Adjusted model	
	Median	iNPH, n (%)	OR (95% CI)	P value	OR (95% CI)	P value
<14.0	12.1	7 (23.3%)	Reference		Reference	
14.0 -16.9	15.5	16 (53.3%)	3.755 (1.239–11.385)	*P* = 0.019	5.360 (1.341–21.427)	*P* = 0.018^*^
≥ 16.9	20.0	18 (60.0%)	4.929 (1.612–15.071)	*P* = 0.005	6.055 (1.501–24.433)	*P* = 0.011^*^

*Adjusted for age, male, former smoking, hypertension, diabetes mellitus, previous stroke, ASCVD/hypercholesterolemia, triglyceride and eGFR levels

## Discussion

This study provides the a comprehensive assessment of the relationship between serum homocysteine and iNPH in DPA. The current study shows a potentially increased risk of iNPH in DPA patients with higher serum homocysteine level after adjusting for a series of potential confounders.

While the symptomatology of iNPH is typical, the pathogenesis and pathophysiology of iNPH remain unclear. The most frequently encountered etiology of iNPH is related to abnormal cerebrospinal fluid dynamics [4]. According to the etiology, cerebrospinal fluid is diffused into the subarachnoid space by the pulsations of intracranial arteries. Each arterial pulsation, a process of attenuation of the pulse wave in the artery, is accompanied by the absorption of cerebrospinal fluid. Therefore, chronic disturbance of arterial pulsation leads to malabsorption of cerebrospinal fluid, resulting in increased intracranial pressure, ventricular enlargement, and hydrocephalus. Elevated homocysteine levels can reduce the compliance of intracranial arteries, due to the toxic effect on the arterial wall [11–13], resulting in reduced arterial pulsation hydrocephalus through the above mechanisms.

There are also other pathogenesis that may explain the relationship between homocysteine and iNPH. Previous studies have proved that elevated homocysteine levels may lead to disruptions of endothelial function through a series of mechanisms, including redox imbalance and oxidative stress resulting in increased protein, nucleic acid and carbohydrate oxidation and lipoperoxidation [12, 14, 27, 28]. The BBB is composed of endothelial cells, and endothelial dysfunction can lead to BBB disruption, increasing BBB permeability [5, 15]. BBB dysfunction has been shown to be associated with iNPH [29]. Moreover, one study showed that iNPH subjects have a 3–4 times higher net CSF volumetric flow rate through the cerebral aqueduct, as compared to reference subjects. In light of the above studies, we speculate that the dynamic disequilibrium of macromolecular transport across the BBB results in an abnormal osmotic gradient between the ventricular system and the vascular system, driving water molecules from the vascular system into the ventricular system and evolving into hydrocephalus. In addition, the endothelial dysfunction would lead to microenvironmental disorders, blood flow imbalance and the obstruction of interstitial drainage fluid, which may lead to hydrocephalus. Our results suggest that there is an association between homocysteine and iNPH, and we speculate that homocysteine may increase the risk of iNPH through the mechanism of reduced the compliance of intracranial arteries or endothelial injury. However, due to the cross-sectional design of the studies and the reference subject being DPA, it remains unclear whether DPA is a cause, effect, or secondary process of iNPH. Further studies with healthy reference subjects are needed.

An increasing number of studies have found an association between homocysteine and individual components of CSVD, such as lacunes [8, 30, 31], CMBs [32], WMH [30–33], enlarged PVS [32] and brain atrophy [8, 34]. It has been suggested that elevated homocysteine levels may lead to CSVD via endothelial dysfunction and subsequent BBB leakage [30], which is similar to the mechanism of the iNPH mentioned above. In our study, the median of homocysteine level was > 15 mmol/L, and higher homocysteine levels were associated with the presence of lobar CMBs and total CMBs, confirming the association between homocysteine and CSVD.

In addition, this study found that age and ASCVD or hyperlipidemia were significantly associated with iNPH, which was consistent with the findings of previous studies on risk factors for iNPH [6, 20]. Currently, aging is considered to be the most relevant risk factor for iNPH, and it is suggested that age-related impairment of meningeal lymphatic CSF drainage and glymphatic fluid exchange, and age-related sleep disorders are the pathogenic mechanism of ventricular enlargement in iNPH [6]. Vascular risk factors, i.e. ASCVD and hyperlipidemia, may cause endothelial dysfunction, increase vascular permeability and disrupt the BBB, leading to ventricular enlargement in iNPH [20, 35]. In contrast to our study, other reports suggest that hypertension, diabetes, obesity, psychosocial factors, and obstructive sleep apnoea are risk factors [6, 20, 21, 35].

The study has several limitations. First, a limitation of the study is the lack of proper matching of patients with controls. Second, due to the limitations of cross-sectional design of the studies, we could not investigate causality. Third, this was a two-center study from southern China, which limits the generalisability of the results. Fourthly, the reference subject was DPA. As mentioned above, DPA, one of the most common forms of CSVD, is associated with elevated homocysteine levels, so our observations may be due to simple coincidences stemming from individuals with a higher burden of CSVD. However, neuroimaging markers and CSVD burden scores were not significantly different between DPA with iNPH and DPA without iNPH in our study. The final limitation is the relatively small sample size, which leaves the possibility of selection bias. Therefore, future multicenter prospective studies with healthy reference subjects are needed to address these issues.

In conclusion, the present study showed a correlation between high serum homocysteine levels and iNPH in DPA; however, further investigation is needed to determine the predictive value of homocysteine and to confirm the underlying mechanism between homocysteine and iNPH.

## Data Availability

Datasets generated and analysed are available from corresponding author upon request.
