# Correction: High homocysteine is associated with idiopathic normal pressure hydrocephalus in deep perforating arteriopathy: a cross-sectional study

**DOI:** 10.1186/s12877-023-04171-y

**Published:** 2023-09-06

**Authors:** Shisheng Ye, Kaiyan Feng, Yizhong Li, Sanxin Liu, Qiaoling Wu, Jinwen Feng, Xiaorong Liao, Chunmei Jiang, Bo Liang, Li Yuan, Hai Chen, Jinbo Huang, Zhi Yang, Zhengqi Lu, Hao Li

**Affiliations:** 1 Department of Neurology, Maoming People’s Hospital, Maoming, China; 2 Department of Radiology, Maoming People’s Hospital, Maoming, China; 3 Department of Neurology, The Third Affiliated Hospital of Sun Yat-sen University, Guangzhou, China; 4 Department of Neurology, Maoming Maternal and Child Health Hospital, Maoming, China

**BMC Geriatrics (2023) 23:382**

10.1186/s12877-023-03991-2

After publication of this article [1], the authors reported that In the Funding section the grant number relating to High-level Hospital Construction Research Project of Maoming People’s Hospital, Maoming Science and Technology Special Fund Plan and Project was incorrectly given as ‘201116164553189’ and should have been ‘2020KJZX006’.

The original article [1] has been corrected.
